# Cone Beam X-Ray Luminescence Tomography Imaging Based on KA-FEM Method for Small Animals

**DOI:** 10.1155/2016/6450124

**Published:** 2016-10-27

**Authors:** Dongmei Chen, Fanzhen Meng, Fengjun Zhao, Cao Xu

**Affiliations:** ^1^College of Life Information Science and Instrument Engineering, Hangzhou Dianzi University, Hangzhou 310018, China; ^2^School of Life Sciences and Technology, Xidian University, Xi'an 710071, China; ^3^School of Information Sciences and Technology, Northwest University, Xi'an, Shaanxi 710069, China

## Abstract

Cone beam X-ray luminescence tomography can realize fast X-ray luminescence tomography imaging with relatively low scanning time compared with narrow beam X-ray luminescence tomography. However, cone beam X-ray luminescence tomography suffers from an ill-posed reconstruction problem. First, the feasibility of experiments with different penetration and multispectra in small animal has been tested using nanophosphor material. Then, the hybrid reconstruction algorithm with KA-FEM method has been applied in cone beam X-ray luminescence tomography for small animals to overcome the ill-posed reconstruction problem, whose advantage and property have been demonstrated in fluorescence tomography imaging. The* in vivo* mouse experiment proved the feasibility of the proposed method.

## 1. Introduction

X-ray luminescence tomography (XLT) has been put forward as a novel imaging technology for biological imaging application based on X-ray-excitable phosphor nanoparticles [1]. These phosphor nanoparticles can produce visible luminescence light signals irradiated with X-ray which can be measured by charge coupled device (CCD) [1]. The discovery that both X-ray and visible light can propagate through tissue and that the nanophosphors agents can trace specific molecular makes XLT a proper tool for* in vivo* biomedical imaging. Nowadays, XLT technology has also been extended from narrow beam X-ray [1] to cone beam X-ray excitation [2] and is even applied in endoscopic imaging [3]. Meanwhile, with advanced specific materials for X-ray excitation, it has also been applied in small animal* in vivo* imaging [4].

The XLT modality has its unique features over other optical imaging methods such as bioluminescence and fluorescence imaging [5, 6]. It can excite the nanophosphors from different angles and avoid a significant autofluorescence in other optical imaging methods [2]. However, this technology demands long scanning time under X-ray exposure, which limits its development of fast* in vivo* biology processes [5]. Some research groups have improved the XLT imaging time resolution from different ways. Carpenter et al. proposed a limited-angle X-ray luminescence tomography method to complete reconstruction from limited-angles in narrow beam XLT system [7]. Badea et al. invented a battleship sampling paradigm to mix sampling and reconstruction in narrow beam XLT system [8]. In addition, Chen et al. designed a cone beam XLT imaging system [2] and Chen et al. put forward a reconstruction method with single view data in cone beam XLT system [3]. Even though the imaging time in the reconstruction method with single view data is reported to be less than 30 s [3], reconstructed result with planar detectors and a single view is generally insufficient for accurate 3D reconstruction [9]. The inverse problem of reconstruction is an ill-posed problem and can be improved by taking images of the experiment subject from multiple views [10] and using multispectral data [11].

It is reported that Gd_2_O_2_S: Tb has several peaks in the spectrum excited by X-rays and can be applied in multispectral imaging and reconstruction [12]. The multispectral property of this material has also been reported for its advantage in improving the XLT imaging quality [13]. However, the penetration property of this material under different spectral has not been discussed before. In this paper, the spectral property of the material in different tissues has been studied. The feasibility of conducting experiments in small animal has been proved through the preliminary results. The hybrid reconstruction algorithm with Kirchhoff approximation and finite element method (KA-FEM) has been studied in fluorescence tomography to overcome the ill-posedness in reconstruction [14]. Hence, we realize cone beam XLT reconstruction with KA-FEM method and perform experiments on both tissue and* in vivo* mouse. Our reconstructed results show that the KA-FEM method can be applied in reconstruction to make XLT imaging feasible for small animal imaging.

## 2. Method

In the cone beam XLT system, X-rays are emitted from the X-ray source and travel through the tissues based on Lambert-Beers' law. Once the nanophosphors are irradiated by X-rays, they will emit visible light. The light transport in biological tissues can be accurately modeled by diffusion approximation, owing to the highly scattering and weakly absorbing properties of the soft tissues in the spectral region. The imaging model can be expressed as follows:

(1) Xr=X0exp⁡−∫r0rμtτdτ,Sr=εXrρr,−∇·Dr∇Φr+μarΦr=Srr∈Ω,

where *X*_0_ is the X-ray source intensity with the initial position **r**_0_ and *μ*_*t*_(*τ*) is the X-ray attenuation coefficient at position *τ* that can be computed from X-ray transmission data using an attenuation-based computed tomography (CT) technique. *S*(**r**) is the light source. *X*(**r**) is the X-ray intensity, and *ρ*(**r**) is the nanophosphor density at position **r**. *ε* is the light yield, while **r** is the position vector. *Ω* is the domain under consideration, and *D*(**r**) = (3(*μ*_*a*_(**r**) + (1 − *g*)*μ*_*s*_(**r**)))^−1^ is the diffusion coefficient with *μ*_*a*_(**r**) as the absorption coefficient, *g* as the anisotropy parameter, and *μ*_*s*_(**r**) as the scattering coefficient. Φ(**r**) is the photon flux density.

Meanwhile, it is reported that the resolution of the reconstructed results can be significantly improved using data measured at different wavelengths [15, 16]. With the surface data measured at two or more wavelengths, the significantly different system matrixes can be obtained to enhance the resolution of the problem. The ratio of the energy distribution in every spectrum can be measured and the linear relationship between the measured multispectral data Φ^meas^ and the material distribution *q* can be obtained as follows:

(2) $$\begin{array}{c} Aq=\Phi^{meas}, \end{array}$$

where

(3) A=ωλ1Aλ1ωλ2Aλ2⋮ωλmAλm,Φmeas=Φλ1Φλ2⋮Φλm,∑ν=1mωλν≈1.

*A*_*λi*_, Φ_*λi*_, and *ω*_*λi*_ represent the system matrix, the measurable photon density, and the relative fraction at which the wavelength *λi* contributes in the emission spectrum, respectively. *ω*_*λi*_ is given as follows:

(4) $$\begin{array}{c} \omega_{\lambda i}=\frac{\int_{\lambda_{i}^{lo}}^{\lambda_{i}^{up}}\xi \left(\lambda \right)d\lambda }{\int_{0}^{\infty }\xi \left(\lambda \right)d\lambda }, \end{array}$$

where *λ*_*i*_^lo^ and *λ*_*i*_^up^ denote the lower and upper limits of the bandpass filter centered on wavelength *λi*, respectively, and *ξ*(*λ*) is the emission spectrum.

The KA-FEM method is applied to combine the analytical method and numerical method based on finite element method to solve the imaging model and form the system equations [14]. The flow chart of the reconstruction can be divided into the KA module and the FEM module. KA method is utilized to produce the region of interest (ROI) and then FEM is used to reconstruct the final result. In the KA module, the system matrix *A*_*λi*_ can be expressed as *G* ^KA^(**r**_**s**_, **r**, ***λ***_**i**_) as follows at the corresponding wavelength ***λ***_**i**_ [14, 17]:

(5) GKArs,r,λi=grs,r,λi+∑p=1Ngrp,r,λi+2CndDλi∂grp,r,λi∂np∂GKArs,rp,λi∂np×ΔSrp,

where ∂**n**_**p**_ denotes the outward normal vector at surface point **r**_**p**_. Δ*S*(**r**_**p**_) denotes local planar discrete area on the surface. *g* denotes Green's function in infinite medium while *G* ^KA^ denotes Green's function in medium with boundary. The surface values ∂*G* ^KA^(**r**_**s**_, **r**_**p**_, ***λ***_**i**_)/∂**n**_**p**_ can be obtained by the method of images [18]:

(6) ∂GKArs,rp,λi∂np=−gR,Z,λi−gR,Z+CndDλiCndDλi,

where **Z** = (**r**_**s**_ − **r**_**p**_)·(−**n**_**p**_) and **R** = **Z** − (**r**_**s**_ − **r**_**p**_). The coefficient *C*_nd_ takes into account the refractive index mismatch between both media [19]. Then, preliminary reconstructed results can be obtained by *l*_1_-norm regularization method, while in the FEM module the system matrix *A*_*λi*_ can be obtained by transforming the model to its weak form and discretizing the domain with the shape function and is expressed as follows at the corresponding wavelength *λi* [2]:

(7) $$\begin{array}{c} A_{\lambda i}=\left(\;{M_{\lambda i}}^{-1}F\;\right)\cdot \varepsilon \cdot X\cdot q, \end{array}$$

where

(8) Mi,j=∫ΩDλi∇Ψi·∇Ψj+μaλiΨi·Ψjdr+12κ∫∂ΩΨiΨjdr,Fi,j=∫ΩΦrΨiΨjdr,Xi,j=Xi,jr.

*ε* is the light yield and *X*_*i*,*j*_(**r**) is the X-ray intensity at each vertex. *κ*(**r**, *n*, *n*′) is the boundary mismatch factor, which depends on the refractive indices *n* in *Ω* and *n*′ in the surrounding medium. Ψ_*i*_ and Ψ_*j*_ denote the corresponding elements of the test function. Finally, the reconstructed result can be obtained with conjugate gradient least square (CGLS) method [20].

## 3. Experiment and Result

**Figure 1:**
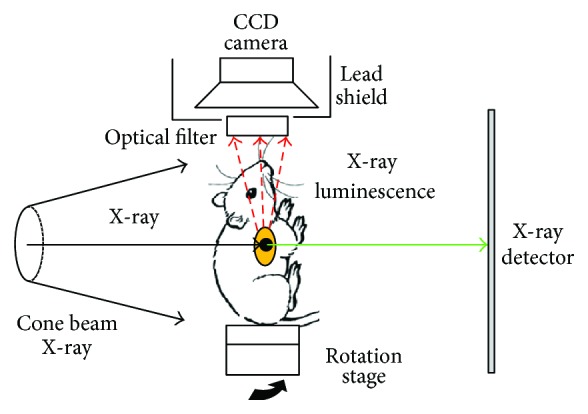
Experimental setup for XLT.

The equipment used in our experiments consisted of a cone beam X-ray source to excite the phosphors, an electron-multiplying CCD (EMCCD) camera to sample the photon fluence, and an extra CMOS X-ray detector panel to collect the transmitted X-rays. This schematic is shown in Figure 1. In the system, the CT system contains a microfocus X-ray source (Apogee, Oxford Instruments, UK) and CMOS flat-panel detector (C7921, Hamamatsu, Japan) with pixel size of 50 *μ*m covering a 1056 × 1056 digital image matrix. The EMCCD camera (PIXIS2048, Princeton Instruments, UK) was mounted at 90° toward the X-ray axis with a Nikkor 55-mm f/2.8 D lens (Nikon, Melville, New York). To minimize the X-ray ionizing radiation to the EMCCD, a lead shield with depth of 4 mm was used.

**Figure 2:**
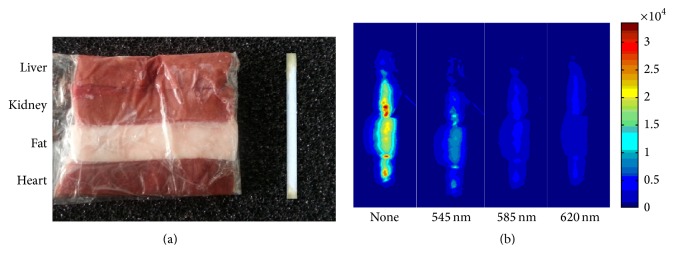
(a) Slices of different porcine tissues (including liver, kidney, fat, and heart) and the material in the plastic capillaries; (b) slice images under different filters.

First the depth of penetration and spectral property of our material were investigated. The experiment was performed by using porcine tissues, including liver, kidney, fat, and heart as shown in Figure 2(a). The fresh porcine tissues were frozen at −20°C and then cut into slices of various thicknesses using a microtome with approximately 10 mm in width and 40 mm in length. The thicknesses of the porcine tissues were from 2 to 6 mm, with a 2 mm interval. The material is put into the plastic capillaries with approximately 2 mm in diameter and 40 mm in length as shown in Figure 2(a). The distance between the material and the X-ray source was 140 mm while the distance between the material and EMCCD camera was 285 mm. The capillaries were placed under the porcine tissues of various thicknesses, including 2, 4, and 6 mm. The X-ray source was set to 50 kVp voltage and 1 mA current. The X-ray luminescent photons emitted from the material were acquired by the CCD camera. A 2 × 2 binning operation was employed to improve the signal to noise ratio (SNR). During the luminescence signal collection, the exposure time was set to 3 s. The image acquisition system was enclosed in a light-tight environment to avoid the effect of light from outside. The proper optical filters with corresponding wavelengths have been selected based on the optical spectra reported in [12]. The optical signals were imaged at wavelengths of 545, 585, and 620 nm with corresponding optical filter manually. The experiment was conducted three times to ensure the accuracy of the measurements.

The luminescent images were obtained as shown in Figure 2(b). The experiment showed that the material can emit visible light signals at wavelengths of 545, 585, and 620 nm corresponding to the spectrum obtained in [12]. The signals are too weak to detect in our system for the slices with depth of 8 mm. The optical images of the material at different depths and different spectra were acquired and the average of the luminescence flux density in ROI is calculated.  Table 1 shows the calculation results of different thickness from different tissues at the wavelength of 620 nm. The color bar in Figure 2(b) and the results of Table 1 show the gray level of the image. Hence, the unit is ADU (Analog to Digital Unit), which can be ignored in both Figure 2(b) and Table 1.  Table 1 shows that the optical signal intensity decreased with the increase in depth. Preliminary experimental results indicated that the penetration depth was different in various porcine tissues and that the optical signals for the material at a depth of 6 mm could be detected for about 3 g material shown in Figure 2(b). The results prove that this material can be applied in the mouse experiment where the sizes of tissues are relatively small; however, we cannot give the precise measurement including all the tissues such as bones and lung under present conditions. Hence, due to the limitation of the material and system, the radius of the mouse selected in the following experiment is about 20 mm and the sizes of tissues are smaller than 6 mm, which is in the range of the detectable depth.

**Figure 3:**
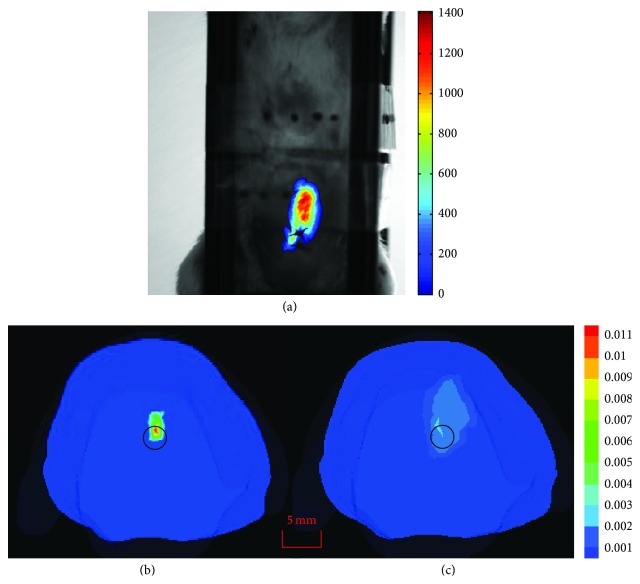
(a) Optical image of* in vivo* small mouse; (b) sectional view of the results with proposed method; (c) sectional view of the results with traditional method.

Then, an* in vivo* mouse experiment was carried out in the above imaging system to evaluate the proposed cone beam X-ray luminescence computed tomography strategy. To evaluate the performance of the proposed method, we implanted a plastic capillary with a 1 mm radius and 2 mm height, which was filled with the Gd_2_O_2_S: Tb nanophosphor, into the mouse with the depth of about 10 mm, to simulate tumor applications. The proper optical filters with corresponding wavelengths have been selected the same as in the tissue slices. The distance between the material and the X-ray source was 200 mm while the distance between the material and EMCCD camera was 270 mm. The micro-CT scanning was also performed (50 kVp, 1.0 mA, 360 views with 1° intervals) in the experiment to get the physical structure and the corresponding X-ray attenuation coefficient of the mouse. Then, the X-ray luminescent material was excited by an X-ray source from four different directions and the luminescent photons emitted from the phantom were acquired by the CCD camera as shown in Figure 3(a). During the luminescence signal collection, the exposure time was set to 5 s for each wavelength separately. The optical signals were imaged at wavelengths of 545, 585, and 620 nm with a binning factor of 2. Due to the limitation of the X-ray source, the total scanning time was about 60 s. The image acquisition system was enclosed in a light-tight environment to avoid the outside light effect.

In the XLT reconstruction, the mouse was discretized into 31189 tetrahedral-elements and 6341 nodes, from the micro-CT results by AMIRA. We compared our method with traditional XLT *l*_1_ regularization method [2] to validate the effectiveness of our proposed method. In the comparison, two benchmarks were applied to evaluate the reconstruction results, including location error and the dice coefficient [21]. The homogeneous absorption coefficient and reduced scattering coefficient were used in the reconstruction processes for three corresponding wavelengths [22]. For the micro-CT information, the reconstruction was performed using the filtered back projection (FBP) method [23]. From the measured data, the distribution of the luminescent material could be reconstructed by the above mentioned method. The center of the capillary in the mouse was 21.2, 21.5, or 13 mm and was obtained from the micro-CT reconstruction result. Figure 3(a) shows the experimental surface data of the mouse. The color bar in Figure 3(a) shows the gray level of the image, whose unit is ADU (Analog to Digital Unit) and is ignored. Figure 3(b) shows the sectional view of the results with proposed method while Figure 3(c) shows the sectional view of the results with traditional method in which the black circle shows the actual material source. Figure 3(b) shows that the proposed method can better reveal the contour of the actual source than the traditional one. From Table 2, it can be seen that the proposed method can achieve smaller location error and larger dice coefficient than the traditional one, which means that the reconstruction results are more similar to the actual source in the proposed method. Both the location error and the dice coefficient in the results can prove that the proposed method can alleviate the ill-posedness and achieve good imaging quality. However, the light yield *ε* of the material could not be obtained in our present system conditions. Hence, the quantity reconstruction results were not discussed and will be studied in future. Therefore, the unit of Figures 3(b) and 3(c) was ignored.

## 4. Discussion

Based on the above experiments, we have demonstrated that the proposed cone beam X-ray luminescence tomography imaging based on KA-FEM method is available to* in vivo* small animal imaging. The comparison between the proposed method and the traditional one shows that the proposed method can alleviate the ill-posedness and achieve good imaging quality from both the location error and the dice coefficient. Even though the spatial resolution of the reconstruction is relatively lower than that of the narrow beam XLT [1], the scanning time in our method could be shortened within 60 s with four angle scanning procedures while the reconstruction can be improved by multispectral imaging. However, the time resolution can be improved if the distance between the material and the X-ray source become smaller with larger FOV of the micro-CT system. The time resolution can also be improved by using a larger voltage or current of the X-ray source within less than 30 s.

The method applied in [13] has also applied multiwavelength information and multilevel mesh strategy in the reconstruction. However, the structural information has been used as important information to realize the quantitative analysis, so the reconstruction error in [13] is smaller than the proposed one. If the reconstruction is conducted without the structural information, the FEM-FEM can achieve the location error of 1 mm and the dice coefficient of 0.38, which is at the same level with the proposed one. Hence, both of them can improve the quality of XLT imaging. However, the KA module in our proposed method can realize reconstruction directly with CT voxel data. This can avoid the tedious segmentation and gridding work in traditional XLT imaging and improve the efficiency of the data process and analysis. Due to the limitation of the personal computer, we cannot deal with the over-large number of the CT voxel data and realize the reconstruction, even though it may be solved in the future with the development of computer science. As a novel imaging technology, the imaging depth of XLT is limited by the material, system, and reconstruction method. It can be improved by increasing optical signal excited by increasing the tube voltage and current of X-ray source, using larger content of material with higher light yield. The influence of these related aspects is a significant task for our further study. Meanwhile, we apply homogeneous model in reconstruction with multispectral data in* in vivo* experiment. It can be deduced from Figure 2 that heterogeneous model for XLT reconstruction may be reasonable because of the difference in optical parameters of different tissues. Unfortunately, in our* in vivo* experiment, a plastic capillary filled with the Gd_2_O_2_S: Tb nanophosphor was implanted into abdomen of the mouse, which may cause large artifacts in CT image and make it difficult to realize the accurate tissue segmentation. With an imprecise tissue segmentation, the reconstruction error of heterogeneous model is much larger than that of homogeneous model. The limitation of tissue data and tissue segmentation cannot fully support the heterogeneous reconstruction in our XLT experiment. With improvement of the segmentation technique and experiment conditions, we will further conduct the heterogeneous reconstruction to study the tissue difference in XLT imaging.

## Figures and Tables

**Table 1:** Calculation results of different thicknesses from different tissues at the wavelength of 620 nm.

	2 mm	4 mm	6 mm
Liver	252.36	18.58	6.95
Kidney	618.96	75.77	27.84
Fat	713.06	272.33	121.79
Heart	420.17	94.15	59.83

**Table 2:** Reconstruction result of the two methods.

	Location error (mm)	Dice coefficient
Proposed method	1.1 mm	0.4
Traditional XLT method	1.8 mm	0.1
